# Case report: The lesson from opioid withdrawal symptoms mimicking paraganglioma recurrence during opioid deprescribing in cancer pain

**DOI:** 10.3389/fpain.2023.1256809

**Published:** 2023-09-22

**Authors:** Elena Ruggiero, Ardi Pambuku, Mario Caccese, Giuseppe Lombardi, Ivan Gallio, Antonella Brunello, Filippo Ceccato, Fabio Formaglio

**Affiliations:** ^1^ Pain Therapy and Palliative Care with Hospice Unit, Veneto Institute of Oncology IOV—IRCCS, Padua, Italy; ^2^ Department of Oncology, Oncology Unit 1, Veneto Institute of Oncology IOV-IRCCS, Padua, Italy; ^3^ Department of Medicine DIMED, University of Padova, Padova, Italy; ^4^ Endocrine Disease Unit, University-Hospital of Padova, Padova, Italy

**Keywords:** cancer pain, opioid, opioid withdrawal, cortisol, catecholamines, opioid deprescribing

## Abstract

Pain is one of the predominant and troublesome symptoms that burden cancer patients during their whole disease trajectory: adequate pain management is a fundamental component of cancer care. Opioid are the cornerstone of cancer pain relief therapy and their skillful management must be owned by physicians approaching cancer pain patients. In light of the increased survival of cancer patients due to advances in therapy, deprescription should be considered as a part of the opioid prescribing regime, from therapy initiation, dose titration, and changing or adding drugs, to switching or ceasing. In clinical practice, opioid tapering after pain remission could be challenging due to withdrawal symptoms’ onset. Animal models and observations in patients with opioid addiction suggested that somatic and motivational symptoms accompanying opioid withdrawal are secondary to the activation of stress-related process (mainly cortisol and catecholamines mediated). In this narrative review, we highlight how the lack of validated guidelines and tools for cancer patients can lead to a lower diagnostic awareness of opioid-related disorders, increasing the risk of developing withdrawal symptoms. We also described an experience-based approach to opioid withdrawal, starting from a case-report of a symptomatic patient with a history of metastatic pheochromocytoma-paraganglioma.

## 1. Introduction

The International Association for the Study of Pain defines pain as “an unpleasant sensory and emotional experience associated with actual or potential tissue damage or described in terms of such damage” (1). However, pain is a personal experience, influenced by psychological and social factors, requiring a personalized and individual approach that often goes beyond guidelines and definitions.

Most of patients with cancer experience pain as the predominant symptom during their illness and cancer treatment, thus affecting the quality of life (QoL). More than half of patients report the presence of moderate-severe pain since cancer diagnosis: its prevalence increases up to 80% in the advanced stage of the disease (2, 3). Cancer pain represents one of the main determinants of QoL and performance status, causing significant limitation in the activities of daily living. Adequate pain management, with pharmacological and non-pharmacological strategies, is therefore essential to preserve QoL and optimize tolerance to life-prolonging treatments, without the need for dose reduction (2–4).

The correct assessment and quantification of pain are essential in the management of cancer patients. Cancer pain treatment is based on the expert use of non-steroidal anti-inflammatory drugs (NSAIDs), opioid, and adjuvant drugs. NSAIDs are indicated for mild to moderate pain, and their side effects are common and relevant in clinical practice. The use of opioid drugs is indicated in the treatment of patients with moderate to severe pain. At each step, the use of adjuvant drugs should be considered (5–7). When opioid drugs have to be prescribed, it must always be taken into consideration the pharmacokinetic and pharmacodynamic bases of tolerance and dependence development. Furthermore, the onset of side effects should be prevented: the patient must always be informed immediately.

As World Health Organization (WHO) suggests, a tiered and personalized approach is needed. The use of non-pharmacological pain management techniques is of utmost importance, to optimize the clinical response. The psychologist should be involved as an active part of the treatment, concurrent with the start of pharmacological therapy. Patients on opioid therapy should be adequately screened for the risk of developing Opioid Use Disorders (OUD). It is of utmost importance to differentiate physical dependence from addiction because psychological vulnerability is relevant in patient with cancer. The onset of withdrawal symptoms after a rapid reduction in opioid dosage, or because of the administration of an antagonist, defines physical dependence. As a precaution, patients should always be considered physically dependent after regular treatment with an opioid drug. Contrariwise, addiction is a syndrome characterized by the loss of control over the use of drugs and by compulsive and continuous use. This is not a pharmacological property of opioid and must be distinguished from physical dependence (5–7).

Although the literature shows a clear increase in the survival of cancer patients, there are still very few studies that have investigated the risk of OUD and withdrawal syndrome in patients with cancer, in which it is possible to plan a tapering or even a cessation of the relief-pain therapy. While there are many studies and clear guidelines on the choice and management of pain therapy in patients with cancer, to date there are no defined guidelines on the tapering of therapy in these patients and on the management of withdrawal symptoms in patients who do not develop addiction.

The clinical case presented allowed us to reflect on the thin red line between the symptoms that may indicate a disease recurrence and those related to opioid tapering, focusing our attention on the need for adequate tools to monitor patients during the deprescribing process. Therefore, the purposes of the article are to highlight the clinical skills and challenges related to opioid therapy in cancer pain, and to suggest how the lack of validated tools for the cancer patient represents a limitation in the process of prescription and deprescription.

## 2. Case presentation: opioid withdrawal mimics a symptomatic recurrence in a patient with metastatic pheochromocytoma-paraganglioma

In April 2010, a 38-year-old female underwent left trans-parotid cervicotomy after the discovery of a left parapharyngeal neoformation. Histology confirmed the diagnosis of vagal paraganglioma. Oncological staging with total body computed tomography (CT) scan and 18F-FDG positron emission tomography did not show distant metastases, urinary catecholamine levels were normal. Therefore, the patient started a clinical and radiological follow-up. Pheochromocytomas and paragangliomas are rare endocrine neoplasms, composed of chromaffin cells, characterized by particular clinical manifestations (due to often catecholamines secretion), and often with a benign outcome after surgery. They are grouped together in the same pheochromocytoma and paraganglioma syndrome (PPGL) (8).

**Figure 1:**
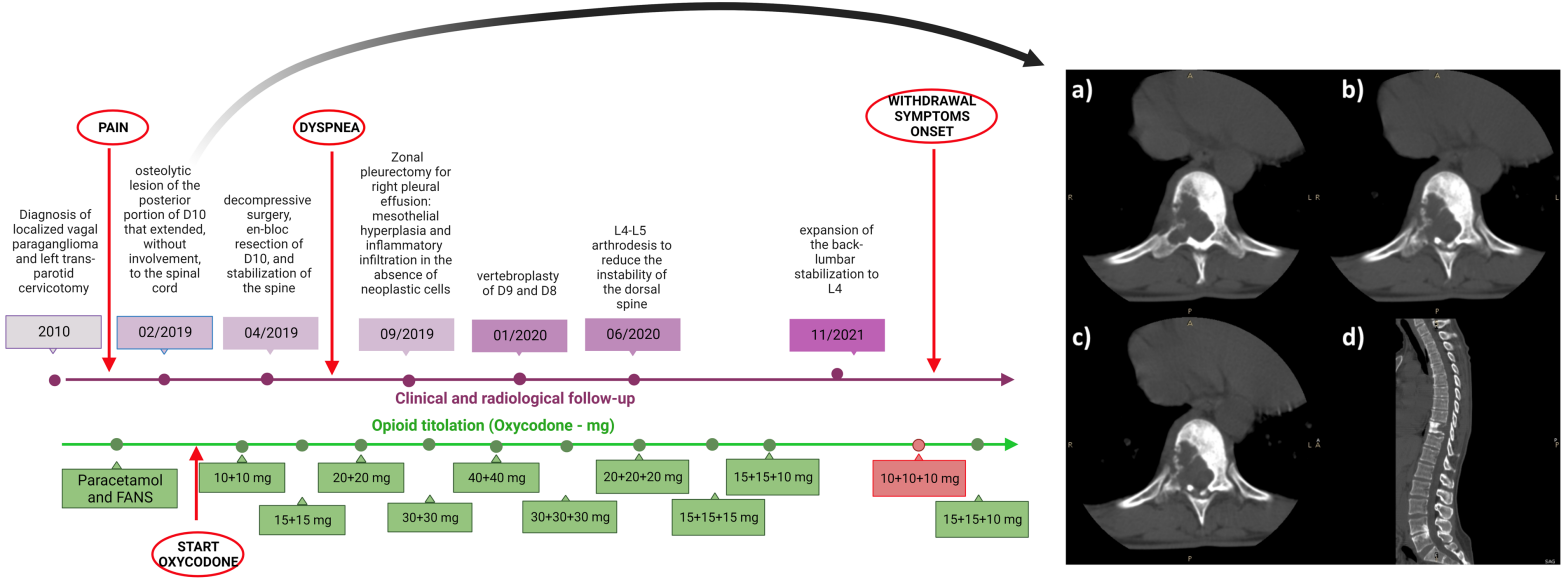
Timeline depicting the clinical history of the patient (in purple) and pain management (in green). On the right: osteolytic lesion of the posterior portion of thoracic vertebra (D10). Panel (**A–C**) Axial plane; panel (**D**) sagittal plane.

In February 2019 she reported back pain (Numeric Pain Rating Scale, NPRS 8/10), therefore she underwent a total spine CT scan with evidence of an osteolytic lesion of the posterior portion of D10 (depicted in Figure 1) that extended, without involvement, to the spinal cord. A biopsy of the D10 osteolytic lesion was performed and confirmed paraganglioma vertebral metastasis.

In April 2019 the patient underwent decompressive surgery, *en bloc* resection of D10, and stabilization of the spine. After this procedure, the patient continued radiological and orthopedic follow-up without finding new-onset metastases. A genetic testing (with next-generation sequencing detection technology) excluded pathogenic mutations. The back pain due to osteolytic lesion was unresponsive to NSAIDs, therefore she started oxycodone 10 mg BID and gabapentin 300 mg BID from May 2019 (the timeline of the treatment is depicted in Figure 1).

The oxycodone dosage was progressively increased according to the patient’s reported NPRS, until the highest dose of 30 mg TID plus rescue therapy with oxycodone/paracetamol 20 mg/325 mg, with benefit on the symptom.

In September 2019 she complained dyspnea: a CT scan revealed right pleural effusion. Histology confirmed the presence of mesothelial hyperplasia and inflammatory infiltration in the absence of neoplastic cells, after a right zonal pleurectomy. A vertebroplasty of D9 and D8 in January 2020 and subsequent L4-L5 arthrodesis in July 2020 were performed to reduce the instability of the dorsal spine. Finally, in November 2021 she underwent an expansion of the back-lumbar stabilization to L4 for the persistence of pain and a sensation of sagging of the lumbar spine, with a reduction on the reported symptom. Opioid treatment was not changed before and after surgical procedures.

She started a progressive monthly tapering of the oxycodone dosage from August 2022, reducing the dosage by about 10%–15% at each step, until reaching the minimum dosage of 10 mg TID in December 2022 with the aim of continuing the progressive reduction of the dosage based on the referred pain (titration and tapering of oxycodone is detailed in Supplementary Table S1). The gabapentin dosage was maintained.

Although there had been no resurgence of pain, suddenly, after about 7–10 days from the reduction from 15 + 15 + 10 mg per day to 10 mg TID, she developed episodes of incoming hypertensive crises: average arterial pressure was 150/80 mmHg, with hypertensive peaks up to 200/100 mmHg especially at night. She also reported palpitations and profuse sweating.

Furthermore, at outpatient clinical evaluation, the patient presented with anxiety, increased respiratory rate, stomach cramps, tachycardia until 100–110 beats per minute, anorexia, and nausea. The symptoms described could- be associated with a secreting recurrence of the paraganglioma (despite normal urinary fractionated catecholamines and metanephrines levels, measured with high-performance liquid chromatography and UV detector: normetanephrine 0.16 μmol/24 h, range 0.01–2.13; metanephrine 0.1 μmol/24 h, range 0.01–1.62; adrenaline 17 nmol/24 h, range 5–110; noradrenaline 141 nmol/24 h, range 40–600). Therefore, therapy with doxazosin 2 mg TID was started, and oxycodone therapy was re-boosted at 15 + 15 + 10 mg.

After the increase of the oxycodone dosage and the introduction of doxazosin, the symptoms rapidly disappeared, with a return to normal blood pressure values after a week (average arterial pressure 120/70 mmHg), without palpitations and sweating, allowing the suspension of the antihypertensive therapy already after about 15 days.

CT scan and 18F-FDG positron emission tomography were performed and excluded a paraganglioma recurrence. The radiological features, combined with clinical data and endocrine evaluation, allowed us to conclude that the symptomatology reported by the patient could be attributed to opioid withdrawal which mimicked a recurrence of secreting paraganglioma.

The rapid disappearance of symptoms with the resumption of oxycodone therapy, the tests performed and the overall clinical picture of the patient led us to exclude other possible causes of hypertension, palpitations and sweating, such as peri-menopausal hormonal variations. After the excluding of recurrent disease, it was agreed with the patient to maintain the dosage of oxycodone until now. A new tapering effort will be planned using a premedication with *α*^2^ agonists (Clonidine).

## 3. Pain management in cancer: the role of opioid

Opioid are the cornerstone of cancer pain relief therapy. Opioid therapy should be administered at different stages of the disease, according to the clinical presentation (9). In choosing the most appropriate therapy, the characteristics of each opioid should be tailored to the clinical features of the individual patient. Pharmacokinetics, pharmacodynamics, adverse events, toxicity, and drug interactions should be considered before treatment. As regards the patients, we must consider gender, age, the possible presence of genetically correlated alterations, site of the primary tumor and metastases, pain characteristics (duration, onset, irradiation, and so on), and any allergies and intolerances, co-morbidities, simultaneous oncological and non-oncological treatments, and excretory organ function (10).

As suggested by the guidelines of the different scientific societies (European Association for Palliative Care, American Society of Clinical Oncology, European Society for Medical Oncology, and WHO) and their most recent revisions, pain-relief therapy and opioid prescribing is based on the WHO steps scale, since 1986 (11–16). In particular:

• weak opioids, such as codeine and tramadol, are recommended if pain is reported as mild to moderate, there are no substantial differences in the choice of drug to start with.
• strong opioids (i.e., morphine, and oxycodone) are recommended when the pain intensity is moderate to severe.

For many years, morphine was considered the first-choice opioid for the treatment of moderate to severe cancer pain. The availability of new molecules and different routes of administration has raised the question of opioid choice. In 2007, a Cochrane Review (17) investigated the role of oral morphine for cancer pain treatment, including 3,749 patients in 54 studies. The high heterogeneity of study design (thirteen and six studies compared modified-release or immediate-release morphine with other opioid respectively, and the others compared different morphine formulations) and the low number of patients recruited in each trial (fewer than 100 participants) were not able to detect a superiority between the formulations or comparative drugs. An updated Cochrane Review confirmed these results: similar efficacy on analgesia and side-effects in opioid-naive patients were reported for oral morphine, oxycodone, and hydromorphone. In clinical practice, several opioid drugs are regularly prescribed in patients with cancer pain: oral morphine (18), oxycodone, or fentanyl (19–23). Only a few differences have been identified across the opioid investigated for the treatment of cancer-related pain, and none of the investigated agents offered a safer profile in terms of adverse events (24).

The choice of the most appropriate opioid for the treatment of moderate or severe cancer pain should balance pharmacokinetic properties (first bioavailability) and the route of administration. Furthermore, in cases of organ failure or renal/kidney impairment, clinicians should try to avoid complications and toxicities. Unless there are no alternatives, morphine should be avoided in case of moderate to severe renal impairment. Oxycodone, fentanyl, and hydromorphone should be carefully monitored because they are primarily excreted in urine (17). A systematic review in 2022 evaluated the use of opioid in patients with cancer and hepatic impairment. The results, conducted in less than a hundred patients, were not sufficient to indicate a preferred opioid in cancer patients with liver dysfunction (25).

According to guidelines, opioid therapy should be initiated as immediate-release formulations and used first on demand for symptom control, at the lowest dose to achieve acceptable analgesia, that can be shared with the patient’s expectations (12, 24). After adequate titration, extended-release or long-acting opioid formulation should be provided with around-the-clock dosing in all patients, with the supply of a rescue short-acting medication to manage breakthrough or transient pain exacerbations (12, 26, 27).

For better pain relief, patients who have been taking other analgesics, such as NSAIDs, may continue these analgesics for a limited time after opioid initiation, if these agents provide additional analgesia and are not contraindicated (24). In addition, for neuropathic pain in advanced cancer patients with opioid failure, the combination with an adjuvant effective in neuropathic cancer pain (GABA-inhibitors, duloxetine, tricyclic antidepressants) has to be considered (5).

### 3.1. Opioid side effects

The most common side effects of long-term opioid administration are constipation, nausea and vomiting, sedation and dizziness, physical tolerance and/or dependence, and respiratory depression. Less common adverse events are delayed gastric emptying, immunologic and hormonal dysfunction (especially regarding cortisol secretion), muscle rigidity and myoclonus, and opioid-induced hyperalgesia. Among them, opioid-induced constipation and nausea are challenging symptoms: they often persist because tolerance does not develop, especially the former. Opioid-induced side effects may be severe enough to require a dose reduction or drug discontinuation (28).

Tolerance, a loss of analgesic efficacy, could lead to progressive increasing dose requirements. Tolerance can be divided into two main classifications:

• Innate: the predisposition genetically determined, that starts from the first opioid dose;
• Acquired: the consequence of repeated drug exposure and linked to pharmacokinetics and pharmacodynamics (29).

Moreover, physical dependence and addiction could prevent proper prescribing and inadequate pain management (28). Pain relief therapy could be insufficient to control symptoms in some opioid-seeking patients; therefore, the term pseudo-addiction has been used to indicate when the clinical presentation is secondary to pain under-treatment of pain, rather than addiction (30).

#### 3.1.1. Opioid-induced constipation (OIC)

The prevalence of OIC ranges from 40% to 95% of opioid-treated patients: its consequences increase morbidity and mortality, with a significant QoL reduction. Finally, long-term chronic constipation can also result in rectal pain, burning, hemorrhoid formation until bowel obstruction with potential bowel rupture, and death (31).

Three subtypes of opioid receptors are reported: *μ*, *δ*, or *κ*. Analgesia is mainly achieved through stimulation of central *μ* receptors; however, opioid receptors are the natural ligands for endogenously produced neurotransmitters in the central and peripheral nervous systems. Opioid activates also those *μ* receptors in the gastrointestinal tract that control gut motility (32, 33). Their activation results in opioid-induced adverse gastrointestinal effects: increased sphincter tone, reduced secretions and increased water absorption, reduced gastric emptying, and reduced propulsion of chime through the intestine (34).

Prevention and treatment of constipation are essential for the management of opioid treatment: the combination with prophylactic laxatives is recommended early, also before the opioid prescription. The two most used central opioid receptor antagonists are naloxone and naltrexone. The former acts also in the peripheral nervous system, it can be administered separately from opioid medications, or combined in fixed-dose tablets. A significant first-pass metabolism explains the predominant intestinal effect of naloxone, explaining its role in treating OIC (35). On the contrary, the Peripherally Acting μ Opioid Receptor Antagonists (PAMORAs) are developed to selectively block peripherally located μ opioid receptors, with minimal effects on the centrally mediated analgesic properties (36).

#### 3.1.2. Opioid-induced nausea and vomiting (OINV)

The activation of opioid receptor in central and peripheral sites leads to OINV (37). The OINV impact on treatment adherence often leads to inadequate pain management; a persistent long-term OINV negatively impacts patient's functional outcomes, well-being, and mental health (38). Several individual factors, such as age, sex, race, genetic polymorphisms, and metabolic differences in pharmacodynamics/pharmacokinetics can explain the large inter-individual variability of OINV onset (39). The incidence of nausea and vomiting after opioid treatment is lower in geriatric patients, while females may have a 60% higher risk of OINV (39, 40).

#### 3.1.3. Opioid-induced hyperalgesia (OIH)

In the long-term management of opioid analgesic therapy, opioid-induced hyperalgesia (OIH) is a paradoxical opioid effect and should not be forgotten: OIH often leads to the need to reduce or modify the therapy (41). OIH is defined as a state of nociceptive sensitization caused by opioid exposure. It is a paradoxical, excessive, and disproportionate pain response in a patient receiving opioid pain therapy, and therefore non-painful stimuli could be perceived negatively by the patient (42).

Treating OIH is often a time-consuming clinical challenge. The main available options include opioid treatment discontinuation or switch to a different opioid, or a low-dose start due to incomplete cross-tolerance, allowing an overall dose reduction. Improved analgesia after opioid rotation has been reported in several clinical situations (43). The use of NMDA antagonists, able to prevent opioid tolerance, is used to manage OIH (44); but there are no large randomized controlled trials and data are not robust. An approach that combines different drugs, as pregabalin and COX-2 inhibitors, may play a role in OIH management (45).

## 4. Opioid use disorders (OUD)

Substance use disorders are chronic illnesses characterized by relapse and remission: OUD definition includes tolerance and withdrawal (46). A checklist of symptoms developed by the American Psychiatric Association is defined in the Diagnostic and Statistical Manual of Mental Disorders, 5th Edition (DSM-5) is used to diagnose OUD (47). It represents an epidemiological emergency, associated with increased mortality, sharply increasing in recent years: an estimated 10.3 million people in the United States misused opioid in 2018, including 9.9 million people who misused opioid for pain treatment (47). Vulnerability to OUD can be affected by several innate and acquired factors, such as genetic background, prolonged exposure to *μ*-opioid agonists for analgesia, untreated underlying psychiatric disorders, young-er age, and social/familial background (48).

OUD assessments proposed by The American Society of Addiction Medicine are structured around six dimensions: acute intoxication, medical conditions and complications, emotional and cognitive conditions or complications, readiness for change, continued use or continued problem potential, recovery/living environment.

### 4.1. Glossary

#### 4.1.1. Abstinence

The intentional and consistent restraint from the pathological pursuit of reward and/or relief. Abstinence involves the use of substances and other behaviors. The term abuse is not recommended for clinical or research purposes, it was previously applied to psychoactive substance-related disorders in the DSM.

#### 4.1.2. Addiction

It is the inability to stop a substance. It is defined as a chronic, relapsing disorder, characterized by compulsive drug seeking and use despite adverse harmful consequences. Addiction is a chronic medical condition that combine the interactions among brain circuits, genetic background, environment, and an individual’s life experiences.

#### 4.1.3. Dependence

Physical dependence is a “state of neurological adaptation that is manifested by a drug class-specific withdrawal syn-drome, produced by abrupt cessation, rapid dose reduction, decreasing blood level of the drug, and/or administration of an antagonist”

- psychological dependence is a “subjective sense of need for a specific psychoactive substance, either for its positive effects or to avoid negative effects associated with its abstinence”.

#### 4.1.4. Tolerance

The reduced responsiveness to an opioid agonist that occurs with its long-term use. Accelerated metabolism and excretion are defined as metabolic tolerance. On the contrary, the central nervous system is less sensitive to the agonist in functional tolerance.

**Figure 2:**
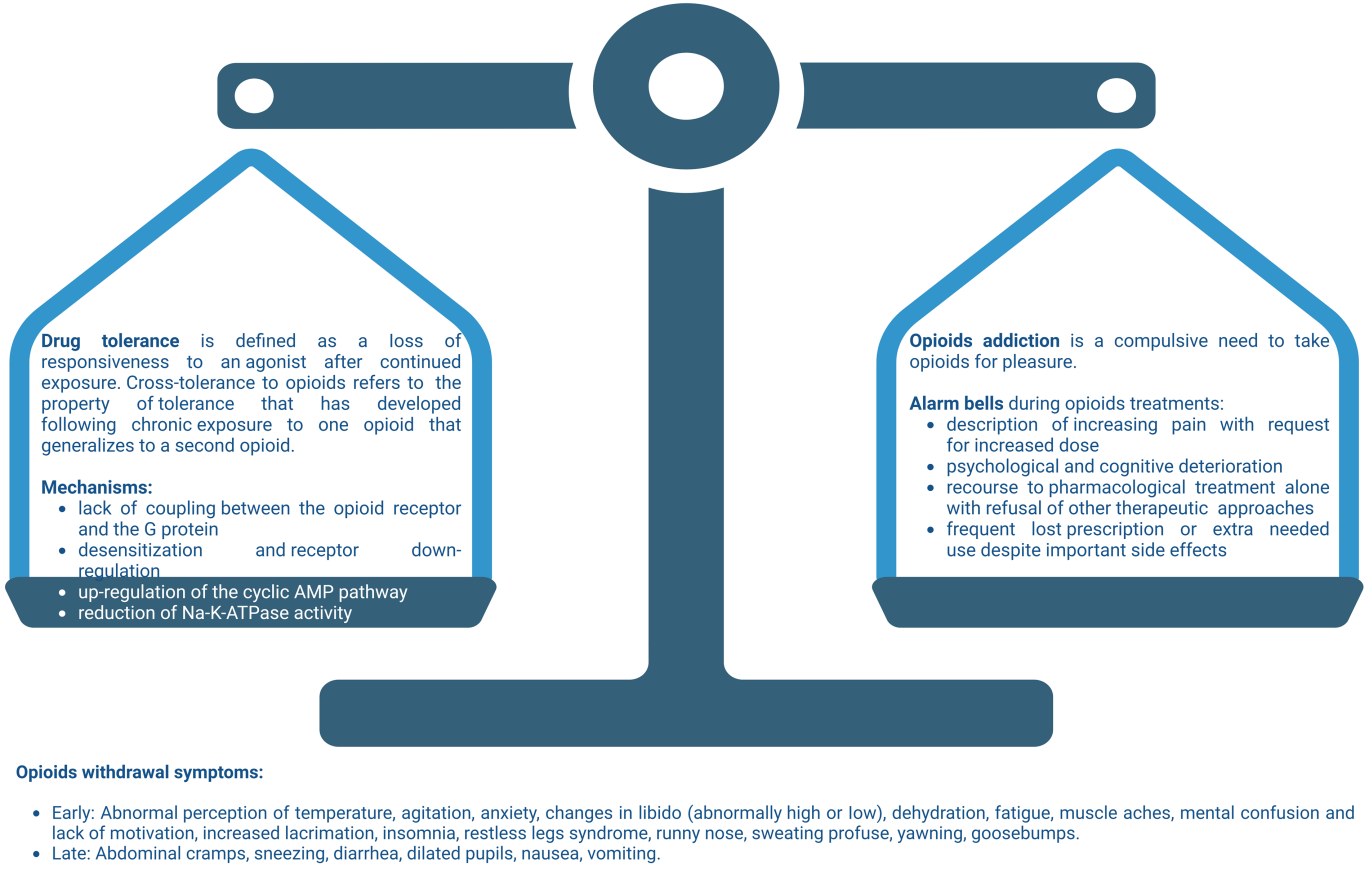
Balance among opioid tolerance and addiction. On the one hand we have the physiological mechanisms of tolerance and physical dependence on opioid. On the other hand, the alarm bells that every doctor must keep in mind during pain-relieving therapy with opioid. Not paying adequate attention to the risk factors which, even in the patient with cancer, can lead to addiction, causes the balance between the two aspects of opioid therapy to become unbalanced. A careful anamnesis and the close monitoring of the patient, allows the physician to keep the two pans of the scales in balance, obtaining the maximum pain response and minimizing the risks. Created with BioRender.com.

Opioid withdrawal begins when the agonistic activity of opioid receptor is reduced after the metabolism of the last dose, after drug discontinuation or antagonist therapy. Withdrawal syndrome is characterized by somatic and psychological symptoms. [reassumed in Figure 2].

## 5. Opioid withdrawal in clinical practice

According to the DSM-5 and as defined in the previous section, withdrawal from a substance is defined as “the substance-specific problematic behavioral change, with physiologic and cognitive components, that is due to the cessation of, or reduction in, heavy and prolonged substance use” and in the International Classification of Diseases, 10th edition, as “a group of symptoms of variable clustering and severity occurring on absolute or relative withdrawal of a psycho-active substance after persistent use of that substance” (49). In clinical practice, common signs and symptoms of opioid withdrawal syndrome are hypertension, tachycardia, nausea, vomiting, diarrhea, mydriasis, piloerection, lacrimation, rhinorrhea, and insomnia (50, 51).

It is generally accepted that a gradual reduction should not be accompanied by the appearance of withdrawal symptoms, while these are to be expected with more robust decreases (52). The drug half-life is one of the main features that affect the onset (early or delayed) opioid withdrawal and the duration of the clinical syndrome. The mechanism of withdrawal, especially regarding the interactions between opioid and noradrenergic systems, explains the symptoms (53, 54). In clinical practice, some common symptoms (such as sedation, hypotension, and reduced respiration rate) are secondary to the suppressed norepinephrine release induced by the μ receptor opioid cAMP-mediated activation on noradrenergic neurons in the locus coeruleus (53, 55, 56). In chronic opioid use, this pathway regains, leading to a norepinephrine excess in the locus coeruleus, which underlies the characteristic symptoms of opioid withdrawal. The μ-opioid receptor agonists and partial agonists (as methadone and buprenorphine) and α2 agonists (clonidine and lofexidine) are used to treat opioid withdrawal symptoms.

### 5.1. Evaluation scales for withdrawal syndrome

There are many scales, tools, and questionnaires that could be useful in the diagnosis and during follow-up of opioid withdrawal syndrome, such as:

• Objective Opioid Withdrawal Scale (OOWS) (57),
• Clinical Opioid Withdrawal Scale (COWS) (58),
• Slubjective Opioid Withdrawal Scale (SOWS) (59).

None of these tools has been specifically validated for the patient with cancer pain.

### 5.2. Feasible pharmacological treatment of withdrawal syndrome

Evidence-based recommendations are lacking: randomized controlled trials are not available to establish which drug is the most suitable for the management of withdrawal symptoms during opioid reduction or withdrawal, particularly in cancer patients (50).

Drug choice strongly depends on the expected goal. Buprenorphine is probably the treatment of choice if the target is the discontinuation of opioid therapy in patients still complaining of pain, given its superiority in terms of pain control over *α*^2^ agonists. However, buprenorphine alone does not control withdrawal symptoms, and *α*^2^ agonists can be combined with adjuvant drugs.

Methadone and buprenorphine are both recommended for the management of opioid withdrawal, their reduction of withdrawal symptoms and improvement of opioid abstinence are similar. Methadone is contraindicated in case of acute bronchial asthma or hypercapnia, and in known or suspected paralytic ileus. Methadone use should be carefully balanced with adverse events in patients with psychiatric disorders, decompensated liver disease, respiratory insufficiency, or concomitant use of sedative, hypnotic, anxiolytic or other QT-interval affecting drugs. Attention should be paid to the substances that interfere with this cytochrome P450 enzyme, such as anticonvulsants, antiretrovirals, and alcohol (60).

Buprenorphine is a partial *µ* opioid receptor agonist available in different formulations, most of which have been approved by the Food and Drug Administration (FDA) since 2015. Buprenorphine is recommended for the pharmacological treatment of OUD and opioid withdrawal: results are similar to methadone, and superior to lofexidine or clonidine (61–64). Buprenorphine should also be used with caution in patients with current or previous hepatic dysfunction, and in patients who have concomitant alcohol or sedative use, hypnotic, or anxiolytic use disorder. Moreover, buprenorphine use in patients with hypovolemia or severe cardiovascular disease may emphasize its hypotensive effects. Significant medication interactions include alcohol, sedatives and agents that affect CYP3A4 activity (azole antifungals, macrolide antibiotics such as erythromycin, and HIV protease inhibitors).

Alpha-2 adrenergic agonists can be used to treat withdrawal syndrome when patients reduce buprenorphine or methadone. 0.1–0.3 mg of Clonidine every 6–8 h, with a maximum dose of 1.2 mg daily, is used until side effects (mainly arterial hypotension). Clonidine is often combined with other non-narcotic medications targeting specific opioid withdrawal symptoms (as benzodiazepines for anxiety, loperamide for diarrhea, acetaminophen or NSAIDs for pain) (65).

Buprenorphine, in contrast, is less limited in several countries. It was introduced for the first time in the U.S. for the treatment of OUD under the Drug Addiction Treatment Act of 2000 (DATA 2000) (66, 67). It allows buprenorphine prescription in outpatients by any licensed physician after a dedicated training. In the US, regulatory restrictions on the use of opioid agonists (or partial agonists) for the treatment of OUD require new legislation that considers the use of buprenorphine. In 2002, the FDA approved buprenorphine in patients with OUD (66, 67). In a 2017 Cochrane review (68), Gowing collected the available clinical evidence regarding the utility of buprenorphine in the treatment of opioid withdrawal. Authors reviewed 27 studies involving 3,048 individuals; the collected manuscripts compared buprenorphine with clonidine or lofexidine (*n* = 14), buprenorphine with methadone (*n* = 6), or buprenorphine tapering (*n* = 7). Buprenorphine was associated with a lower withdrawal score than *α*^2^ agonists (clonidine and lofexidine, seven studies in 902 patients), longer retention in treatment (five studies, 558 patients), and increased likelihood of withdrawal treatment completion (12 studies in 1,264 cases). A direct comparison among studies was not feasible because each author used a different scale. In such scenario, an effort of the scientific community is to homogenize the scale used. Buprenorphine was similar to methadone in terms of treatment duration or completion rate. Clonidine reduces noradrenergic hyperactivity in locus coeruleus neurons that cause opioid withdrawal symptoms, and therefore is effective in patients (53, 56, 69). In a 2016 Cochrane review, Gowing and colleagues (65) investigated the use of *α*^2^ agonists (especially clonidine and in minor parts lofexidine, tizanidine, and guanfacine) in the treatment of opioid withdrawal. *α*^2^ agonist treatment (five studies with clonidine, one with lofexidine) was more effective than placebo and more likely to result in the completion of treatment, withdrawal signs and symptoms occurred and resolved earlier than methadone, and treatment duration was shorter. Also in this case measures of withdrawal severity differed between studies (50).

## 6. Endocrine aspects of opioid use and withdrawal

Several drugs (not only opioid) are able to modulate the activity of the hypothalamic–pituitary–adrenal (HPA) axis or the sympathetic nervous system, leading to an impaired secretion or activity of respectively cortisol or catechol-amines. Moreover, somatic and motivational symptoms accompanying opioid withdrawal are secondary to the activation of stress-related processes (either cortisol or catecholamines).

The impact of chronic opioid administration and its final result in HPA axis tolerance-induced and abstinence-induced ACTH/cortisol hypersecretion has been extensively studied in animal models. In rats, ACTH and corticosterone (rat steroidogenesis does not secrete cortisol) responses to CRH and ACTH were not related to morphine tolerance (70). In mice, a systemic pretreatment with prazosin or propranolol (the selective antagonists of the *α*^1^-adrenergic and the *β*-adrenergic receptor, respectively), or with spironolactone (the mineralocorticoid receptor antagonist) decreased somatic symptoms of morphine withdrawal (induced with naloxone after a chronic morphine treatment), allowing to measure somatic and affective/motivational aspects of physical morphine dependence. The withdrawal symptoms assessed in the study were jumps, rearing, paw tremors, body tremors, wet-dog shakes, teeth chattering, defecations, and urinations. Moreover, in the same model, only propranolol pretreatment attenuated the dysphoric affective state accompanying opioid withdrawal (71). Lateral hypothalamic orexin system function extends beyond reward seeking: it can play a role in the expression of addiction-like state in rats (72). The locus coeruleus is involved in opioid addiction development: in mice, hypothalamic hypocretin innervation increases after morphine administration, correlated with an increase in tyrosine hydroxylase expression (the enzyme that catalyzes the rate limiting step in this synthesis of catecholamines). Elimination of hypocretin neurons prevents the tyrosine hydroxylase increase and reduces the somatic and affective components of opioid withdrawal (73). Depleted animals showed a significant decrease in the global withdrawal syndrome score compared with intact controls after naloxone-precipitated withdrawal (73). C1 neurons in the rostral ventrolateral medulla express the adrenaline-synthesizing enzyme phenylethanolamine N-methyltransferase: chronic morphine use in rats produces a selective internalization of mu-opioid receptors in C1 neurons, and may precipitate the sympathetic hyperactivity during acute opioid withdrawal (74).

Finally, it has been speculated that some opioid can be quickly converted to catecholamines *in vivo*. It is suggested to occur in addition to the acute conversion into their known metabolites, and can play a role in abuse and dependence. Not only analgesic opioid (morphine and oxycodone) but also their antagonists (naltrexone and naloxone) may be converted to catecholamines through a series of currently unidentified reactions. From a chemical point-of-view, the molecule of morphine and oxycodone contain some domains (especially methyl groups): the formation of dopamine or epinephrine is possible if these methyl groups are enzymatically removed (75).

In humans, chronic opioid dependence may cause the altered function of the HPA axis, and opioid withdrawal may change cortisol or amine concentrations. In 2008 it has been reported a decreased pituitary responsiveness and an increased adrenal cortisol response in two groups of patients with opioid dependence treated with benzodiazepines and clonidine (30 patients), or with methadone (30 patients). CRH concentration during acute abstinence was lower in patients treated with benzodiazepines and clonidine or methadone; CRH levels then normalized 30 and 90 days after withdrawal in the former group. Contrariwise, ACTH levels were similar to controls. Cortisol levels during acute abstinence were higher in patients treated with benzodiazepines and clonidine and lower in those treated with methadone (both with respect to controls) (76). Metyrapone test is considered convenient and sensitive, when compared with the insulin-induced hypoglycemia, to assess the integrity of HPA axis function (77). The metyrapone test was performed in 18 methadone-maintained former heroin addict patients, 10 without and 8 with ongoing cocaine dependence, resulting in ACTH hyperresponsivity in the latter group (78).

In mammals, not only the adrenal cortex (HPA axis) but also the adrenal medulla (with catecholamines) is involved in stress response and may be affected by opioid use and its withdrawal. Plasmatic adrenaline concentrations in-crease up to 30-fold (with a minor magnification of noradrenaline) after the initial injection of naloxone during ultra-rapid opioid detoxification, leading to increased heart rate and stroke volume (79). In the same setting of naloxone receptor blockade for opioid detoxification, pre-administration of the *α*^2^-adrenoceptor agonist clonidine decreases the muscle sympathetic activity and catecholamine plasma concentrations (80). However, a concomitant antisocial personality disorder (ASPD) can induce a possible impairment of this clonidine effect (81).

Some case reports support the evidence that opioid withdrawal leads to sympathetic hyperactivity and increased catecholamine release, which may trigger catecholamine-induced cardiomyopathy, resulting in heart failure and fatal arrhythmias (8). Takotsubo is characterized by transient left ventricular apex wall motion abnormality, similar to acute coronary syndrome but with normal coronary artery flow. In 2021, it has been reported a case of a woman with a past history of intravenous drug abuse on opioid substitution treatment with buprenorphine. She presented in the Emergency Department with tachycardia, tachypnea, and bilateral rales; she reported that she did not use buprenorphine for three days. A takotsubo cardiomyopathy was found in left ventriculography (apical ballooning): opioid withdrawal mediates sympathetic overdrive and may trigger takotsubo syndrome development (82). The main hypothesis for the physiopathology of takotsubo is that a rapid and significant increase of serum catecholamines (secondary to a stressful event: it was also known as the “broken heart syndrome” may cause microvascular coronary spasm, with inflammation and dysfunction (83). The main model of sympathetic overstimulation is pheochromocytoma and its related cardiomyopathy (84).

Clonidine is a central *α*^2^-adrenoreceptors agonist, it reduces sympathetic outflow and noradrenaline release from sympathetic nerve endings. Obviously, autonomous catecholamine secretion from pheochromocytoma-paraganglioma (PPGL) is not affected by clonidine: suppression test with clonidine is used in patients with suspected PPGL and moderate endocrine excess (85).

Evidence-based studies regarding endocrine aspects of withdrawal syndrome are limited in patients that use opioids for addiction, and extremely limited in cancer patients during the withdrawal phase. Catecholamine-based symptoms are only a puzzle piece of the several aspects that must be considered in a holistic approach when facing a patient.

## 7. The opioid deprescribing in cancer pain

In the last decades, cancer-therapy improvements have dramatically changed the natural history of the disease for many patients, modifying their life expectancy from diagnosis and survival curves (86). In this scenario, there are several reasons that may bring out the clinical or social need to reduce or stop opioid pain therapy (87). Pain could reduce or completely remit after an effective cancer cure, either from surgery or radiotherapy, or pharmacological treatments. Sedation and other opioid side effects could obstacles to work and activities resume; indeed, long-term opioid therapies increase risks of abuse and misuse.

**Figure 3:**
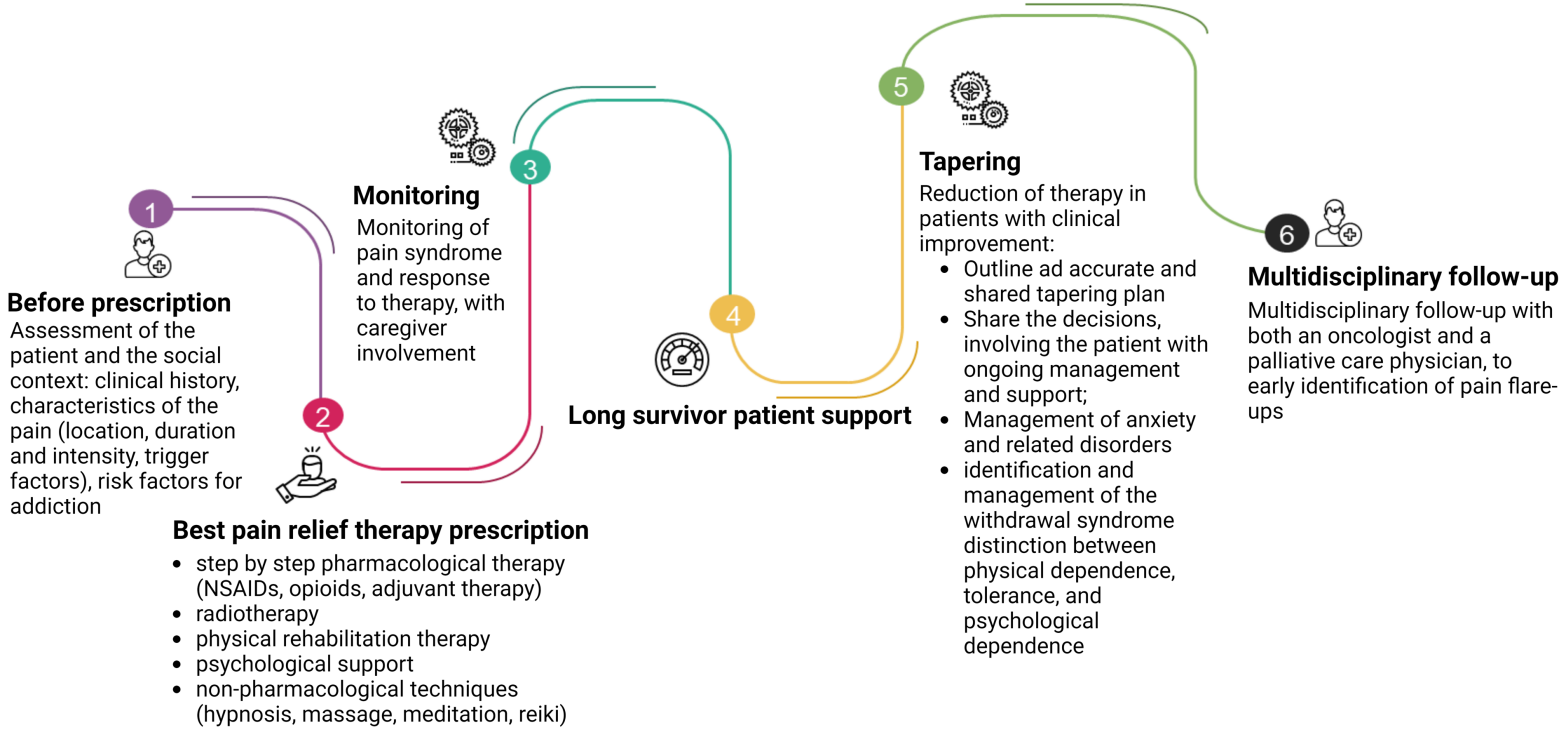
The journey of opioid prescription. At first, before prescription, the physician has to do a thorough assessment of the patient and the social context. The prescription of the pain-relief therapy must follow the guidelines, considering the clinical characteristics of the patient and the localization of the disease. Also, radiotherapy and physical rehabilitation have to be considered. The psychological aspects and non-pharmacological therapies should not be underestimated. Equal attention must be given to the tapering of pain-relieving therapy: each step must be shared with the patient, who has to be carefully monitored and instructed on the symptoms that may appear (i.e., hypertension, tachycardia, sweating, palpitations, …). A multidisciplinary path involving oncologists and pain specialists, shared with the patient, can significantly reduce the risk of developing OUD for the patient, especially in long survivors. Created with BioRender.com.

Deprescription integrates opioid management expertise from the prescribing schedule, to opioid therapy initiation, dose titration, to switching or discontinuation, as reassumed in Figure 3. Opioid tapering with the intention to discontinue when side effects outweigh the benefits must always be considered (87). As already addressed in the previous chapters, protracted opioid use is associated with many adverse effects, such as constipation, nausea and vomiting, daytime somnolence, increased risk of falls, and poor concentration or memory loss. It has also been high-lighted an increased mortality rate in patients taking 100 mg/24 h of morphine or equivalent, compared with doses equivalent low than 20 mg/24 h (87, 88).

However, while the guidelines on the initiation and management of cancer pain relief therapy with opioid are clear, indications for the tapering of opioid therapy in cancer patients are very poor and often developed in the context of drug addiction (89). Alongside the pharmacological strategies previously reported, the literature suggests some fundamental principles that can be the cornerstone of the correct management of this therapeutic phase, reassumed in Table 1.

**Table 1:** Opioid deprescribing process: from risk factor to shared decision.

Opioid deprescribing process in cancer patients: from risk factor to shared decision	
Risk factors of developing analgesic dependence	
- previous personal or family history of addiction,	
- reluctance to acknowledge psychological contributors to pain,	
- significant psychiatric comorbidity	
- social isolation	
Potential obstacles:	Potential facilitator:
- fear of pain recrudescence	- stable family and friendly support
- fear of less effective non-opioid analgesic drugs	- proper education in recognizing the side effects of opioid,
- feeling of abandonment in the deprescribing process,	- identify personal reasons that may affect the route (i.e., work and driving restrictions)
- limited availability and affordability of health care.	- use of non-pharmacological therapies
Key principles of correct management of opioid deprescribing	
1. share the importance of reducing opioid to the patient, according to the phase of the disease and the life-prolonging therapies,	
2. involve the patient with ongoing management and support,	
3. outline ad accurate and shared tapering plan,	
4. management of anxiety and related disorders,	
5. ensure the patient understands the difficulty of the pathway and the need for support.	

Clinicians should always work with patients and their caregivers to define the best approach for pain management, immediately establishing realistic goals, with a view to a shared planning of the therapeutic path (90–92). Furthermore, risk factors for developing analgesic dependence must be seriously considered also for cancer patients, particularly when the disease is in remission. If UOD emerges from the beginning of the therapy in the general population suffering from chronic pain, in cancer patients it is conceivable that the aspects of psychological dependence emerge more clearly in the deprescribing process.

It is therefore essential, in the choice of pain-relief therapy, an accurate clinical history that highlights the potential risk factors of OUD. Furthermore, the role of the psychologist who can follow the patient in the various phases of the disease and also in that of the reduction of opioid therapy is fundamental. These factors may not preclude the use of opioid therapy for pain, but their management needs careful supervision (88).

Proper management of tapering of opioid in cancer patients should be indeed based on five cardinal points (88):

1. Share the importance of reducing opioid with the patient, according to the phase of the disease and the life-prolonging therapies,
2. Involve the patient, giving him as much freedom as possible about how to reduce opioid, with ongoing management and support,
3. Accurate and shared planning of the tapering until the discontinuation,
4. Adopt multidisciplinary strategies for the management of anxiety and related disorders,
5. Ensure the patient understands the difficulty of the pathway and the need for support.

## 8. Conclusions

The clinical case that we presented allowed us to reflect on the underestimated incidence of withdrawal syndrome in cancer patients on long-term opioid therapy. In clinical practice, the symptoms presented by the patient mimicked a cancer recurrence, requiring an appropriate modification of the follow-up times and involving an important amount of anxiety to the patient herself, which a careful management of the opioid deprescribing process would have avoided. Our findings are limited to a single case report, that we used as a springboard for the whole work. It is a limitation, nonetheless larger studies with a sufficient number of patients are required to confirm that clonidine (or other α-agonist) should be used in the withdrawal phase in patients treated with opioid with cancer pain, not only in those with addiction.

In this narrative review, we summarized some aspects that are relevant to the medical management of opioid treatment for cancer pain. First, opioid withdrawal syndrome can mimic several symptoms that are very common in cancer patients. We used a scholarly case presentation of a patient with a history of metastatic sporadic paraganglioma, to underline the endocrine aspects of opioid withdrawal syndrome. Moreover, in cancer patients, a high psychological burden leads patients and physicians to underestimate some distinctive symptoms of abstinence as asthenia and nausea.

The clinical case also allowed us also to highlight the lack of guidelines on opioid tapering in cancer patients. Patients with cancer pain clearly represent a different population from those with chronic non-cancer pain, due to the evolutionary characteristics of the underlying disease, which often lead to long-term therapy. One of the fundamental reflections that emerge from our experience is that the correct timing for reducing therapy must be identified according to the patient, who has to be correctly informed about the symptoms that may appear in this process. As is often done in titration, even in the deprescribing it could be useful to create a “therapeutic diary” for the patient, to report withdrawal symptoms. Follow-up by the pain specialist should also be strictly timed.

In clinical practice, there are several tools that can help physicians to unmask opioid withdrawal; nonetheless, none of them is tailored to the patient that uses opioid to relief cancer pain: an effort in this regard is suggested to the scientific community of palliative specialists. These tools should be validated not only for inpatients, but their use is “out” of the hospitalization, at home, and pain management should be shared with caregivers, nurses, and general practitioners: the awareness of the pain and opioid use (and its withdrawal syndrome) should be enriched in all clinical settings.

The most relevant food for thought that emerges with respect to the management of this clinical case is the frequent lack of education of the patient to promptly recognize withdrawal symptoms. Patients with cancer are used to experiencing negative symptoms related to life-prolonging treatments and therefore resilient towards the appearance of even disturbing symptoms. In developing *ad hoc* guidelines for opioid deprescribing, we deem it useful to suggest the creation of a self-completion questionnaire for daily administration that allows the patient to promptly identify the onset of withdrawal symptoms. It is also essential to illustrate, share and agree from the beginning of opioid tapering on the therapeutic attitude to maintain in the event of the onset of symptoms: return to the previous dosage vs use of drugs for symptom management vs. multimodal non-pharmacological approach. The keyword must therefore be the sharing of decisions. Furthermore, the presence of a multidisciplinary team is the added factor to optimize the patient management, integrating moreover the outpatient approach with home service.

## Data Availability

The original contributions presented in the study are included in the article/**Supplementary Material**, further inquiries can be directed to the corresponding author.

## Supplementary material

Supplementary Table 1 Timeline of oxycodone tapering compared with pain severity.

## References

[1] Raja SN Carr DB Cohen M Finnerup NB Flor H Gibson S The revised international association for the study of pain definition of pain: concepts, challenges, and compromises. Pain. (2020) 161(9):1976–82. 10.1097/j.pain.0000000000001939 32694387 PMC7680716

[2] Caraceni A Shkodra M. Cancer pain assessment and classification. Cancers (Basel). (2019) 11(4):510. 10.3390/cancers11040510 30974857 PMC6521068

[3] van den Beuken-van Everdingen MHJ Hochstenbach LMJ Joosten EAJ Tjan-Heijnen VCG Janssen DJA. Update on prevalence of pain in patients with cancer: systematic review and meta-analysis. J Pain Symptom Manage. (2016) 51(6):1070–90.e9. 10.1016/j.jpainsymman.2015.12.340 27112310

[4] Ruggiero E Tizianel I Caccese M Lombardi G Pambuku A Zagonel V Advanced adrenocortical carcinoma: from symptoms control to palliative care. Cancers (Basel). (2022) 14(23):5901. 10.3390/cancers14235901 36497381 PMC9739560

[5] TERAPIA DEL DOLORE IN ONCOLOGIA—linee guida AIOM. (2021). Available to: https://snlg.iss.it/wp-content/uploads/2022/06/LG-484-AIOM_Dolore.pdf

[6] Caraceni A Martini C Zecca E Fagnoni E. Cancer pain management and palliative care. Handb clin neurol. Amsterdam, The Netherlands: Elsevier (2012). p. 391–415. Available at: https://linkinghub.elsevier.com/retrieve/pii/B978044452138500027X 10.1016/B978-0-444-52138-5.00027-X 22230457

[7] Varrassi G Coluzzi F Guardamagna VA Puntillo F Sotgiu G Vellucci R. Personalizing cancer pain therapy: insights from the rational use of analgesics (RUA) group. Pain Ther. (2021) 10(1):605–17. 10.1007/s40122-021-00248-x 33730338 PMC8119556

[8] Ceccato F Correa R Livhits M Falhammar H. Editorial: predictive tools in pheochromocytoma and paraganglioma. Front Endocrinol (Lausanne). (2023) 14:13–5. 10.3389/fendo.2023.1227543/full PMC10298152 37383393

[9] Mercadante S. Cancer pain treatment strategies in patients with cancer. Drugs. (2022) 82(13):1357–66. 10.1007/s40265-022-01780-6 36129661

[10] Corli O Floriani I Roberto A Montanari M Galli F Greco MT Are strong opioids equally effective and safe in the treatment of chronic cancer pain? A multicenter randomized phase IV ‘real life’ trial on the variability of response to opioids. Ann Oncol. (2016) 27(6):1107–15. 10.1093/annonc/mdw097 26940689

[11] Mestdagh F Steyaert A Lavand’homme P. Cancer pain management: a narrative review of current concepts, strategies, and techniques. Curr Oncol. (2023) 30(7):6838–58. 10.3390/curroncol30070500 37504360 PMC10378332

[12] Fallon M Giusti R Aielli F Hoskin P Rolke R Sharma M Management of cancer pain in adult patients: ESMO clinical practice guidelines. Ann Oncol. (2018) 29:iv166–91. 10.1093/annonc/mdy152 30052758

[13] Aman MM Mahmoud A Deer T Sayed D Hagedorn JM Brogan SE The American society of pain and neuroscience (ASPN) best practices and guidelines for the interventional management of cancer-associated pain. J Pain Res. (2021) 14:2139–64. 10.2147/JPR.S315585 34295184 PMC8292624

[14] WHO Guidelines for the pharmacological and radiotherapeutic management of cancer pain in adults and adolescents. Geneva, Switzerland: World Health Organization (2018). Available at: https://www.ncbi.nlm.nih.gov/books/NBK537492/ (Cited September 7, 2023). 30776210

[15] Caraceni A Hanks G Kaasa S Bennett MI Brunelli C Cherny N Use of opioid analgesics in the treatment of cancer pain: evidence-based recommendations from the EAPC. Lancet Oncol. (2012) 13(2):e58–68. 10.1016/S1470-2045(12)70040-2 22300860

[16] Wiffen PJ Wee B Derry S Bell RF Moore RA. Opioids for cancer pain—an overview of cochrane reviews. Cochrane Database Syst Rev. (2017) 2020(2). 10.1002/14651858.CD012592.pub2 PMC6483487 28683172

[17] Wiffen PJ McQuay HJ. Oral morphine for cancer pain. In: Wiffen PJ, editors. Cochrane database of systematic reviews. Chichester, UK: John Wiley & Sons, Ltd (2007). 10.1002/14651858.CD003868.pub2 17943804

[18] Caraceni A Pigni A Brunelli C. Is oral morphine still the first choice opioid for moderate to severe cancer pain? A systematic review within the European palliative care research collaborative guidelines project. Palliat Med. (2011) 25(5):402–9. 10.1177/0269216310392102 21708848

[19] Schmidt-Hansen M Bromham N Taubert M Arnold S Hilgart JS. Buprenorphine for treating cancer pain. Cochrane Database Syst Rev. (2015) 2018(12). 10.1002/14651858.CD009596.pub4 PMC6513197 25826743

[20] Schmidt-Hansen M Bennett MI Arnold S Bromham N Hilgart JS Page AJ Oxycodone for cancer-related pain. Cochrane Database Syst Rev. (2022) 2022(6). 10.1002/14651858.CD003870.pub7 PMC9180760 35679121

[21] Nicholson AB Watson GR Derry S Wiffen PJ. Methadone for cancer pain. Cochrane Database Syst Rev. (2017) 2017(3). 10.1002/14651858.CD003971.pub4 PMC6464101 28177515

[22] Wang D-D Ma T-T Zhu H-D Peng C-B. Transdermal fentanyl for cancer pain: trial sequential analysis of 3,406 patients from 35 randomized controlled trials. J Cancer Res Ther. (2018) 14(8):14. 10.4103/0973-1482.171368 29578144

[23] Li Y Ma J Lu G Dou Z Knaggs R Xia J Hydromorphone for cancer pain. Cochrane Database Syst Rev. (2021) 2021(8). 10.1002/14651858.CD011108.pub3 PMC8406835 34350974

[24] Paice JA Bohlke K Barton D Craig DS El-Jawahri A Hershman DL Use of opioids for adults with pain from cancer or cancer treatment: ASCO guideline. J Clin Oncol. (2023) 41(4):914–30. 10.1200/JCO.22.02198 36469839

[25] Hughes LT Raftery D Coulter P Laird B Fallon M. Use of opioids in patients with cancer with hepatic impairment—a systematic review. BMJ Support Palliat Care. (2022) 12(2):152–7. 10.1136/bmjspcare-2021-003065 34470772

[26] Klepstad P Kaasa S Borchgrevink PC. Starting step III opioids for moderate to severe pain in cancer patients: dose titration: a systematic review. Palliat Med. (2011) 25(5):424–30. 10.1177/0269216310386280 21708850

[27] Dowell D Ragan KR Jones CM Baldwin GT Chou R. CDC clinical practice guideline for prescribing opioids for pain—United States, 2022. MMWR Recomm Rep. (2022) 71(3):1–95. 10.15585/mmwr.rr7103a1 36327391 PMC9639433

[28] Benyamin R Trescot AM Datta S Buenaventura R Adlaka R Sehgal N Opioid complications and side effects. Pain Physician. (2008) 11(2 Suppl):S105–20. 10.36076/ppj.2008/11/S105 18443635

[29] Collett BJ. Opioid tolerance: the clinical perspective. Br J Anaesth. (1998) 81(1):58–68. 10.1093/bja/81.1.58 9771273

[30] Greene MS Chambers RA. Pseudoaddiction: fact or fiction? An investigation of the medical literature. Curr Addict Rep. (2015) 2(4):310–7. 10.1007/s40429-015-0074-7 26550549 PMC4628053

[31] Swegle JM Logemann C. Management of common opioid-induced adverse effects. Am Fam Physician. (2006) 74(8):1347–54. 17087429

[32] Kurz A Sessler DI. Opioid-induced bowel dysfunction. Drugs. (2003) 63(7):649–71. 10.2165/00003495-200363070-00003 12656645

[33] Herndon CM Jackson KC Hallin PA. Management of opioid-induced gastrointestinal effects in patients receiving palliative care. Pharmacotherapy. (2002) 22(2):240–50. 10.1592/phco.22.3.240.33552 11837561

[34] De Schepper HU Cremonini F Park M-I Camilleri M. Opioids and the gut: pharmacology and current clinical experience. Neurogastroenterol Motil. (2004) 16(4):383–94. 10.1111/j.1365-2982.2004.00513.x 15305992

[35] Meissner W Schmidt U Hartmann M Kath R Reinhart K. Oral naloxone reverses opioid-associated constipation. Pain. (2000) 84(1):105–9. 10.1016/S0304-3959(99)00185-2 10601678

[36] Prichard D Norton C Bharucha AE. Management of opioid-induced constipation. Br J Nurs. (2016) 25(10):S4–11. 10.12968/bjon.2016.25.10.S4 27231750

[37] Smith HS Laufer A. Opioid induced nausea and vomiting. Eur J Pharmacol. (2014) 722:67–78. 10.1016/j.ejphar.2013.09.074 24157979

[38] Nicholson BD. Economic and clinical burden of opioid-induced nausea and vomiting. Postgrad Med. (2017) 129(1):111–7. 10.1080/00325481.2017.1243004 27690715

[39] Cepeda M. Side effects of opioids during short-term administration: effect of age, gender, and race. Clin Pharmacol Ther. (2003) 74(2):102–12. 10.1016/S0009-9236(03)00152-8 12891220

[40] Suh D-C Kim MS Chow W Jang E-J. Use of medications and resources for treatment of nausea, vomiting, or constipation in hospitalized patients treated with analgesics. Clin J Pain. (2011) 27(6):508–17. 10.1097/AJP.0b013e31820d9b76 21368666

[41] Sampaio-Cunha TJ Martins I. Knowing the enemy is halfway towards victory: a scoping review on opioid-induced hyperalgesia. J Clin Med. (2022) 11(20):6161. 10.3390/jcm11206161 36294488 PMC9604911

[42] Lee M Silverman SM Hansen H Patel VB Manchikanti L. A comprehensive review of opioid-induced hyperalgesia. Pain Physician. (2011) 14(2):145–61. 10.36076/ppj.2011/14/145 21412369

[43] Mercadante S Arcuri E. Hyperalgesia and opioid switching. Am J Hosp Palliat Med. (2005) 22(4):291–4. 10.1177/104990910502200411 16082916

[44] Ramasubbu C Gupta A. Pharmacological treatment of opioid-induced hyperalgesia: a review of the evidence. J Pain Palliat Care Pharmacother. (2011) 25(3):219–30. 10.3109/15360288.2011.589490 21834699

[45] Roeckel L-A Le Coz G-M Gavériaux-Ruff C Simonin F. Opioid-induced hyperalgesia: cellular and molecular mechanisms. Neuroscience. (2016) 338:160–82. 10.1016/j.neuroscience.2016.06.029 27346146

[46] Urits I Patel A Zusman R Virgen CG Mousa M Berger AA A comprehensive update of lofexidine for the management of opioid withdrawal symptoms. Psychopharmacol Bull. (2020) 50(3):76–96. 32733113 10.64719/pb.4615 PMC7377538

[47] The ASAM national practice guideline for the treatment of opioid use disorder: 2020 focused update. J Addict Med. (2020) 14(2S):1–91. 10.1097/ADM.0000000000000633 32511106

[48] Kreek MJ Reed B Butelman ER. Current status of opioid addiction treatment and related preclinical research. Sci Adv. (2019) 5(10). 10.1126/sciadv.aax9140 31616793 PMC6774730

[49] WHO. International statistical classification of diseases and related health problems, 10th revision. Geneva, Switzerland: World Health Organization (2016).

[50] Srivastava AB Mariani JJ Levin FR. New directions in the treatment of opioid withdrawal. Lancet (London, England). (2020) 395(10241):1938–48. 10.1016/S0140-6736(20)30852-7 32563380 PMC7385662

[51] Dowell D Compton WM Giroir BP. Patient-Centered reduction or discontinuation of long-term opioid analgesics. JAMA. (2019) 322(19):1855. 10.1001/jama.2019.16409 31600366 PMC7145754

[52] *Guidance for opioid reduction in primary care*. (2020). Available at: Guidance for opioid reduction in primary care—JQ/HW clinical leads (Cited April 15, 2023).

[53] Mazei-Robison MS Nestler EJ. Opiate-induced molecular and cellular plasticity of ventral tegmental area and locus coeruleus catecholamine neurons. Cold Spring Harb Perspect Med. (2012) 2(7):a012070. 10.1101/cshperspect.a012070 22762025 PMC3385942

[54] TR K. Neurobiology of abused drugs. J Nerv Ment Dis. (1990) 178(4):217–27. 10.1097/00005053-199004000-00001 2156952

[55] Kosten T George T. The neurobiology of opioid dependence: implications for treatment. Sci Pract Perspect. (2002) 1(1):13–20. 10.1151/spp021113 18567959 PMC2851054

[56] Kosten TR Baxter LE. Review article: effective management of opioid withdrawal symptoms: a gateway to opioid dependence treatment. Am J Addict. (2019) 28(2):55–62. 10.1111/ajad.12862 30701615 PMC6590307

[57] Peachey JE Lei H. Assessment of opioid dependence with naloxone. Br J Addict. (1988) 83(2):193–201. 10.1111/j.1360-0443.1988.tb03981.x 3345396

[58] Wesson DR Ling W. The clinical opiate withdrawal scale (COWS). J Psychoactive Drugs. (2003) 35(2):253–9. 10.1080/02791072.2003.10400007 12924748

[59] Handelsman L Cochrane KJ Aronson MJ Ness R Rubinstein KJ Kanof PD. Two new rating scales for opiate withdrawal. Am J Drug Alcohol Abuse. (1987) 13(3):293–308. 10.3109/00952998709001515 3687892

[60] Alawa J Muhammad M Kazemitabar M Bromberg DJ Garcia D Khoshnood K Medication for opioid use disorder in the Arab world: a systematic review. Int J Drug Policy. (2022) 102:103617. 10.1016/j.drugpo.2022.103617 35182841 PMC9851143

[61] Ling W Charuvastra C Collins JF Batki S Brown LS Kintaudi P Buprenorphine maintenance treatment of opiate dependence: a multicenter, randomized clinical trial. Addiction. (1998) 93(4):475–86. 10.1046/j.1360-0443.1998.9344753.x 9684386

[62] Oesterle TS Thusius NJ Rummans TA Gold MS. Medication-assisted treatment for opioid-use disorder. Mayo Clin Proc. (2019) 94(10):2072–86. 10.1016/j.mayocp.2019.03.029 31543255

[63] Blanco C Volkow ND. Management of opioid use disorder in the USA: present status and future directions. Lancet. (2019) 393(10182):1760–72. 10.1016/S0140-6736(18)33078-2 30878228

[64] Dematteis M Auriacombe M D’Agnone O Somaini L Szerman N Littlewood R Recommendations for buprenorphine and methadone therapy in opioid use disorder: a European consensus. Expert Opin Pharmacother. (2017) 18(18):1987–99. 10.1080/14656566.2017.1409722 29183228

[65] Gowing L Farrell M Ali R White JM. Alpha 2 -adrenergic agonists for the management of opioid withdrawal. Cochrane Database Syst Rev. (2016):18. 10.1002/14651858.CD002024.pub5 PMC7081129 27140827

[66] Shulman M Wai JM Nunes E V. Buprenorphine treatment for opioid use disorder: an overview. CNS Drugs. (2019) 33(6):567–80. 10.1007/s40263-019-00637-z 31062259 PMC6585403

[67] J JAFFE. From morphine clinics to buprenorphine: regulating opioid agonist treatment of addiction in the United States. Drug Alcohol Depend. (2003) 70(2):S3–11. 10.1016/S0376-8716(03)00055-3 12738346

[68] Gowing L Ali R White JM Mbewe D. Buprenorphine for managing opioid withdrawal. Cochrane Database Syst Rev. (2017) 2017(2). 10.1002/14651858.CD002025.pub5 PMC6464315 28220474

[69] Kleber HD. Pharmacologic treatments for opioid dependence: detoxification and maintenance options. Dialogues Clin Neurosci. (2007) 9(4):455–70. 10.31887/DCNS.2007.9.2/hkleber 18286804 PMC3202507

[70] Ignar DM Kuhn CM. Effects of specific mu and kappa opiate tolerance and abstinence on hypothalamo-pituitary-adrenal axis secretion in the rat. J Pharmacol Exp Ther. (1990) 255(3):1287–95. 2175800

[71] Solecki WB Kus N Gralec K Klasa A Pradel K Przewłocki R. Noradrenergic and corticosteroid receptors regulate somatic and motivational symptoms of morphine withdrawal. Behav Brain Res. (2019) 360(October 2018):146–57. 10.1016/j.bbr.2018.11.041 30500430

[72] James MH Stopper CM Zimmer BA Koll NE Bowrey HE Aston-Jones G. Increased number and activity of a lateral subpopulation of hypothalamic orexin/hypocretin neurons underlies the expression of an addicted state in rats. Biol Psychiatry. (2019) 85(11):925–35. 10.1016/j.biopsych.2018.07.022 30219208 PMC7528037

[73] McGregor R Wu M-F Holmes B Lam HA Maidment NT Gera J Hypocretin/orexin interactions with norepinephrine contribute to the opiate withdrawal syndrome. J Neurosci. (2022) 42(2):255–63. 10.1523/JNEUROSCI.1557-21.2021 34853083 PMC8802943

[74] Drake CT Aicher SA Montalmant FL Milner TA. Redistribution of mu-opioid receptors in C1 adrenergic neurons following chronic administration of morphine. Exp Neurol. (2005) 196(2):365–72. 10.1016/j.expneurol.2005.08.012 16194531

[75] Fitzgerald PJ. Many drugs of abuse may be acutely transformed to dopamine, norepinephrine and epinephrine in vivo. Int J Mol Sci. (2021) 22(19). 10.3390/ijms221910706 PMC8509043 34639047

[76] Zhang GF Ren YP Sheng LX Chi Y Du WJ Guo S Dysfunction of the hypothalamic-pituitary-adrenal axis in opioid dependent subjects: effects of acute and protracted abstinence. Am J Drug Alcohol Abuse. (2008) 34(6):760–8. 10.1080/00952990802385781 19016181

[77] Ceccato F Scaroni C. Central adrenal insufficiency: open issues regarding diagnosis and glucocorticoid treatment. Clin Chem Lab Med. (2019) 57(8):1125–35. 10.1515/cclm-2018-0824 30427776

[78] Schluger JH Borg L Ho A Kreek MJ. Altered HPA axis responsivity to metyrapone testing in methadone maintained former heroin addicts with ongoing cocaine addiction. Neuropsychopharmacology. (2001) 24(5):568–75. 10.1016/S0893-133X(00)00222-0 11282257

[79] Kienbaum P Thurauf N Michel MC Scherbaum N Gastpar M Peters J. Profound increase in epinephrine concentration in plasma and cardiovascular stimulation after [micro sign]-opioid receptor blockade in opioid-addicted patients during barbiturate-induced anesthesia for acute detoxification. Anesthesiology. (1998) 88(5):1154–61. 10.1097/00000542-199805000-00004 9605673

[80] Kienbaum P Heuter T Michel MC Scherbaum N Gastpar M Peters J. Sympathetic neural activation evoked by μ-receptor blockade in patients addicted to opioids is abolished by intravenous clonidine. Anesthesiology. (2002) 96(2):346–51. 10.1097/00000542-200202000-00020 11818767

[81] Gerra G Ceresini S Esposito A Zaimovic A Moi G Bussandri M Neuroendocrine and behavioural responses to opioid receptor-antagonist during heroin detoxification: relationship with personality traits. Int Clin Psychopharmacol. (2003) 18(5):261–9. 10.1097/00004850-200309000-00002 12920386

[82] Laina A Latsios G Dri E Aggeli C Tsiamis E Tsioufis K. Buprenorphine withdrawal induced takotsubo cardiomyopathy: a series of unfortunate events. J Cardiol Cases. (2022) 25(5):316–8. 10.1016/j.jccase.2021.11.012 35582081 PMC9091532

[83] Spadotto V Zorzi A ElMaghawry M Meggiolaro M Pittoni GM. Heart failure due to ’stress cardiomyopathy’: a severe manifestation of the opioid withdrawal syndrome. Eur Hear J Acute Cardiovasc Care. (2013) 2(1):84–7. 10.1177/2048872612474923 PMC3760581 24062938

[84] Wang Y Yu X Huang Y. Predictive factors for catecholamine-induced cardiomyopathy in patients with pheochromocytoma and paraganglioma. Front Endocrinol (Lausanne). (2022) 13. 10.3389/fendo.2022.853878/full PMC8959126 35355563

[85] Remde H Pamporaki C Quinkler M Nölting S Prejbisz A Timmers HJLM Improved diagnostic accuracy of clonidine suppression testing using an age-related cutoff for plasma normetanephrine. Hypertension. (2022) 79(6):1257–64. 10.1161/HYPERTENSIONAHA.122.19019 35378989

[86] Forman D Gatta G Capocaccia R Janssen-Heijnen ML Coebergh JW. Cancer survival. Lancet. (2001) 357(9255):555. 10.1016/S0140-6736(05)71700-1 11229693

[87] Hamilton M Gnjidic D Christine Lin C-W Jansen J Weir KR Shaheed CA Opioid deprescribing: qualitative perspectives from those with chronic non-cancer pain. Res Soc Adm Pharm. (2022) 18(12):4083–91. 10.1016/j.sapharm.2022.07.043 35963766

[88] Heartshorne R. Dose reduction of long-term opioids: our duty as clinicians. Br J Gen Pract. (2019) 69(681):191. 10.3399/bjgp19X701957 30923148 PMC6428460

[89] Sandhu H Underwood M Furlan A Noyes J Eldabe S. What interventions are effective to taper opioids in patients with chronic pain? Br Med J. (2018) 362:k2990. 10.1136/bmj.k2990 30262590

[90] Emery J Butow P Lai-Kwon J Nekhlyudov L Rynderman M Jefford M. Management of common clinical problems experienced by survivors of cancer. Lancet. (2022) 399(10334):1537–50. 10.1016/S0140-6736(22)00242-2 35430021

[91] Paice JA Portenoy R Lacchetti C Campbell T Cheville A Citron M Management of chronic pain in survivors of adult cancers: american society of clinical oncology clinical practice guideline. J Clin Oncol. (2016) 34(27):3325–45. 10.1200/JCO.2016.68.5206 27458286

[92] Koyyalagunta D Bruera E Solanki DR Nouri KH Burton AW Toro MP A systematic review of randomized trials on the effectiveness of opioids for cancer pain. Pain Physician. (2012) 15(3 Suppl):ES39–58. 10.36076/ppj.2012/15/ES39 22786461

