# Supplementary material for: Case report: The lesson from opioid withdrawal symptoms mimicking paraganglioma recurrence during opioid deprescribing in cancer pain
Source: Front Pain Res (Lausanne). 2023 Sep 22;4:1256809. doi: 10.3389/fpain.2023.1256809 (PMC10556467; doi:10.3389/fpain.2023.1256809)
Supplement: Supplementary Table 1 — Timeline of oxycodone tapering compared with pain severity. [file Table1.pdf]

Case report: the lesson from opioid withdrawal symptoms mimicking paraganglioma recurrence during opioid deprescribing in cancer pain

Elena Ruggiero, Ardi Pambuku, Mario Caccese, Giuseppe Lombardi, Ivan Gallio, Antonella Brunello, Filippo Ceccato, Fabio Formaglio

Supplementary Table

DATE	NUMERIC PAIN RATING SCALE	DOSE OF OXYCODONE	Notes
**TITOLATION PHASE**			
**05/2019**	NPRS 8/10	Oxycodone 10mg BID	Add Gabapentin 300mg TID
**06/2019**	NPRS 7/10	Oxycodone15mg BID	
**07/2019**	NPRS7/10	Oxycodone 20mg BID	
**08/2019**	NPRS 6/10	Oxycodone 30mg BID	
**09/2019**	NPRS 7/10	Oxycodone 40mg BID	
**10/2019- 02/2020**	NPRS 3-4/10	no changes in therapy	
**03/2020**	NPRS 7/10	Oxycodone 30mg TID	
**04/2020-07/2021**	NPRS 3-4/10	no changes in therapy	
**TAPERING PHASE**			
**08/2022**	NPRS 3-4/10	Oxycodone 15mg TID	
**09/2022**	NPRS 4/10	Oxycodone 15+15+10mg	
**10/2022**	NPRS 3-4/10	no changes in therapy	
**11/2022**	NPRS 3-4/10	Oxycodone 10 mg TID	Onset of withdrawal symptoms after a week
**12/2022**	NPRS 4/10	Oxycondone 15+15+10 mg	Add Doxazosin 2 mgTID
**01/2023**	NPRS 3-4/10	Oxycondone 15+15+10 mg,	Antihypertensive therapy only as needed
